# The Election of Medical Superintendent of Bethlem Hospital

**Published:** 1852-07-01

**Authors:** 


### THE ELECTION OF MEDICAL SUPERINTENDENT OF BETHLEM HOSPITAL.

Out of twenty-four medical gentlemen who offered themselves as candidates for the office of physician and resident medical superintendent of Bethlem Hospital, the three following gentlemen have been selected by the committee of management as fully qualified to go to the poll.

Robert Jamieson, Esq., M.D. of Aberdeen.
Lockhart Robertson, M.D. (late of the Royal Medical Asylum, Yarmouth.
W. C. Hood, M.D., of Colney Hatch.

Both Dr. Jamieson and Dr. Robertson's antecedents are such as to fully justify their aspiring to the high and distinguished post for which they are candidates. We should regret to say one word calculated to give pain, to wound the feelings, or damage the interest of the other candidate, but having a responsible duty to discharge, and anxious to promote the well-being of the great institution over which the new resident officer will have to preside, we feel called upon, in a public capacity, to point out to those upon whom the election depends, the vital importance of well weighing the qualifications of the three gentlemen from whom the selection is to be made. Dr. Jamieson's testimonials are unquestionably of a high order. His lectures on the *Medical Jurisprudence of Insanity,* published in the "*Medical Gazette,*" fully attests his literary ability. Apart from this, his practical acquaintance with the subjects of mental philosophy, medical psychology, and the actual treatment of the insane, is such as to point him out as a gentleman in every way calculated, to do honour to an institution like Bethlem. The election of Dr. Jamieson would, we are certain, give great and unqualified satisfaction to the medical profession. Dr. L. Robertson's age is said to be adverse to his interests; but he is an able, active, intelligent, and working man, and his nomination to the post will also be gratifying to all who have the pleasure of his personal acquaintance, and who are conversant with his many and varied attainments. The contest, we presume, will *virtually* rest between Dr. Jamieson and Dr. L. Robertson. We sincerely hope that wisdom far superior to that of which man can boast will guide those delegated with the great and solemn responsibility of electing the gentleman into whose hands the future destinies of this great national asylum are to be placed.

